# Diversity of Underwater Vocalizations in Chinese Soft-Shelled Turtle (*Pelodiscus sinensis*)

**DOI:** 10.3390/ani13050812

**Published:** 2023-02-23

**Authors:** Lu Zhou, Jinhong Lei, Xiaofei Zhai, Ningning Lu, Haitao Shi, Jichao Wang

**Affiliations:** Ministry of Education Key Laboratory for Ecology of Tropical Islands, Key Laboratory of Tropical Animal and Plant Ecology of Hainan Province, College of Life Sciences, Hainan Normal University, Haikou 571158, China

**Keywords:** *Pelodiscus sinensis*, vocalization diversity, underwater recordings, call types, age-sex difference

## Abstract

**Simple Summary:**

Vocalization is important for the survival of underwater animals. *Pelodiscus sinensis* spends most of its life in water, and its wild population is highly vulnerable. However, its underwater vocalization, which can serve as the basis for ecological and evolutionary research, has not been explored. We identified 10 call types of the species from underwater recordings and found differences in vocalizations between individuals of different ages and sexes. The Chinese soft-shelled turtle has a high diversity of vocalizations and shows a tendency for vocalization to become more diverse with age. These features help them to adapt to complex and dark underwater environments.

**Abstract:**

Sound communication is important for underwater species. The wild population of the Chinese soft-shelled turtle (*Pelodiscus sinensis*) is listed as vulnerable. However, its vocalization, which can serve as the basis for ecological and evolutionary research, has not been studied. Here, we performed underwater recordings of 23 Chinese soft-shelled turtles of different ages and sexes and identified 720 underwater calls. The turtle calls were manually divided into 10 call types according to visual and aural inspection properties. The similarity test indicated that the manual division was reliable. We described the acoustic properties of the calls and the statistical analysis showed that the peak frequency of calls was significantly different between adult females and males, and also between subadults and adults. Similar to other aquatic turtles that prefer to live in deep water, Chinese soft-shelled turtles have a high vocal diversity and many harmonic calls, indicating that this highly aquatic species developed a variety of vocalizations to enhance their underwater communication, which helped them adapt to the complex and dim underwater environment. Furthermore, the turtles showed a tendency for vocalization to become more diverse with age.

## 1. Introduction

As an indispensable source of information, sound is important for underwater species [1]. Vocalization plays an important role in a turtle’s life, conveying a large amount of individual information [2,3], and is associated with mating and incubation [4,5,6]. Female *Testudo marginata* prefer fast-rate and high-pitched calls in males [5]. Vocalization of *T. hermanni* can transmit information about individuals’ size and health status and is related to their mating success [2]. Moreover, the vocalizations of *Chelonia mydas* and *Eretmochelys imbricata* hatchlings in a nest are related to the synchronization of emergence [4,6].

According to previous studies, turtle vocalizations are relatively simple compared to those of other groups of species, mostly coming in the form of pulses and harmonics [7,8,9,10,11,12]. Early studies on the vocalization of turtles were mainly in the air, such as *Dermochelys coriacea* and *Testudinidae* [3,13,14,15]. However, in recent years, an increasing number of studies have focused on the underwater vocalizations of turtles. *Chelonida oblonga* produces 17 call types in semi-natural pools [10]. Three call types of *Carettochelys insculpta* [16], 11 call types of *Podocnemis expansa* [17], and six call types of *Lepidochelys kempii* [7] were recorded in the field. Cultured *Mauremys sinensis* and *Trachemys scripta elegans* can produce 10 and 12 underwater sounds, respectively [12,18], and even juvenile green turtles (*C. mydas*) in field can produce 11 types of underwater sounds [19].

Researchers believe that reptiles intermittently regulate the passage of air by expulsing large amounts of air and changing the size of the glottis [20]. According to the anatomy of the turtle’s throat, the structure and position of the hyoid cartilage and two bands of elastic fibers in the tortoise’s larynx suggest that these bands are capable of vibrating, and two diverticula supported by the cricoid form a resonating chamber, all of which may function as vocal cords [21]. This may explain why turtles can make underwater sounds.

The most common method for turtle call classification is to record vocalizations and manually classify them according to visual and auditory differences [10,16]. Turtles make sounds when they move underwater in tanks, such as crawling, stroking water, releasing bubbles, sucking water, scratching claws against the tank bottom, and scratching the tank wall with claws, and spectrograms of the sounds produced by these behaviors have been reported [18]. These sounds can be easily distinguished from the vocalizations of turtles [12]. The correlation coefficient test of signals is a traditional method for checking the degree of similarity between two signals [22,23]. A method that combines manual classification with a signal similarity test can decrease the subjectivity of the manual division of calls, and it performed well in a previous study for turtle call classification [12].

Chinese soft-shelled turtles (*Pelodiscus sinensis*) live in many countries in East and Southeast Asia [24]. In China, they are bred in large numbers on farms, but their wild population continues to decline, and it is listed as a “Vulnerable species” by the International Union for Conservation of Nature Red List of Threatened Species [25]. The physiology, ecology, and molecular biology of this species have been extensively studied [26,27,28,29], but its vocalizations have not been studied. The Chinese soft-shelled turtle inhabits inland fresh water and is a highly aquatic turtle (spends most of its life in water). These turtles prefer to live in deep water, normally 1–2 m and they dive into the mud at the bottom of the water most of the time [29]. Thus, underwater vocalizations may be crucial for communication between individuals of the Chinese soft-shelled turtle species. Therefore, vocalization research can provide the basis for the study of the behaviors and ecology of Chinese soft-shelled turtles.

In this study, passive acoustic monitoring was used to investigate the diversity of underwater vocalizations of Chinese soft-shelled turtles in a laboratory. We aimed to classify calls and describe their acoustic characteristics. Moreover, we used a similarity test to verify the objectivity of classification. We also analyzed differences in vocalizations across sex and age groups.

## 2. Materials & Methods

### 2.1. Data Collection

Twenty-three healthy, captive Chinese soft-shelled turtles were used for the recordings: four adult females, four adult males, four subadult females, four subadult males, and seven hatchlings. These turtles live in semi-natural pools in a feedlot. We recorded the five groups of turtles. In addition, eight adult male and female turtles were mixed and recorded to determine whether there were any vocalizations associated with mating. The recordings were performed at Hainan Normal University in July 2021, in a professional soundproof room, free from environmental noise.

Turtles were recorded in an inflatable plastic circular tank, which effectively reduced the sound reflection. The diameter of the tank was 1.2 m, and the water depth was 30 cm. The turtles could move freely through the water, and the hydrophone was placed in the center of the tank, 15 cm below the surface. Vocalizations of turtles were recorded using a Song Meter SM4 underwater sound recorder (Wildlife Acoustics, Inc., Maynard, MA, USA) (omnidirectional hydrophone bandwidth: 2–30,000 Hz ± 3 dB; sensitivity: −165 dB Re: 1 V/μPa; sampling rate: 44,100 Hz; gain: 16 dB). The hydrophone was calibrated by the company (Wildlife Acoustics, Inc.) before purchase. Each group was continuously recorded for 24 h, and 144 h of data were obtained.

### 2.2. Data Analysis

Raven Pro 1.6 software (The Cornell Lab of Ornithology, USA) was used to visualize the waveforms and spectra of the recorded data. Sounds with spectral shapes similar to published turtle sounds [10,12,16] were selected by researchers with experience in turtle vocalizations. Only calls with a high signal-to-noise ratio were selected. Before the selection of turtle calls, we identified the sounds produced by the common behaviors of turtles in water based on studies of other freshwater turtles [12]. Turtle calls were classified based on differences in auditory sensing and spectrograms [10,12]. Two calls that showed extremely similar spectral properties and aural characteristics were classified as the same type. Sennheiser IE300 earphones (Sennheiser, Hanover, Germany) were used to analyze the data. The low frequency (lower limit of a call, Hz), high frequency (upper limit of a call, Hz), peak frequency (frequency of a call at its highest energy point, Hz), signal duration (the time difference between end time and start time of a call, ms), and number of harmonics of calls were extracted using the Raven Pro 1.6 software.

We chose the Pearson correlation coefficient to calculate the similarity between calls to judge the accuracy of the manual division [15,16]. This included the similarity between calls of different call types and similarity between calls of the same call type. Pearson’s linear correlation coefficient [30] was calculated using MATLAB R2021a (MathWorks Inc., Natick, MA, USA).

### 2.3. Statistical Analyses

The median and interquartile range (IQR) for all call types were calculated. We evaluated the differences in two acoustic parameters between age and sex groups: peak frequency and duration. First, the parameters of the sounds for each group were estimated using the Kolmogorov–Smirnov test [31] to determine if they followed a normal distribution. If the distribution was normal (*p* > 0.05), the differences in the parameters of the calls among groups were tested using the least significant difference test (post hoc test algorithms); if not (*p* < 0.05), the differences were tested using Kruskal–Wallis analysis (non-parametric tests algorithms) [32]. Second, if the differences in the parameters of the calls among the groups were significant (*p* < 0.05), paired comparisons were conducted between every two groups to estimate the differences. Differences were considered statistically significant at *p* < 0.05. These analyses were completed using IBM SPSS Statistics 26.0 (IBM, Armonk, NY, USA).

## 3. Results

**Figure 1:**
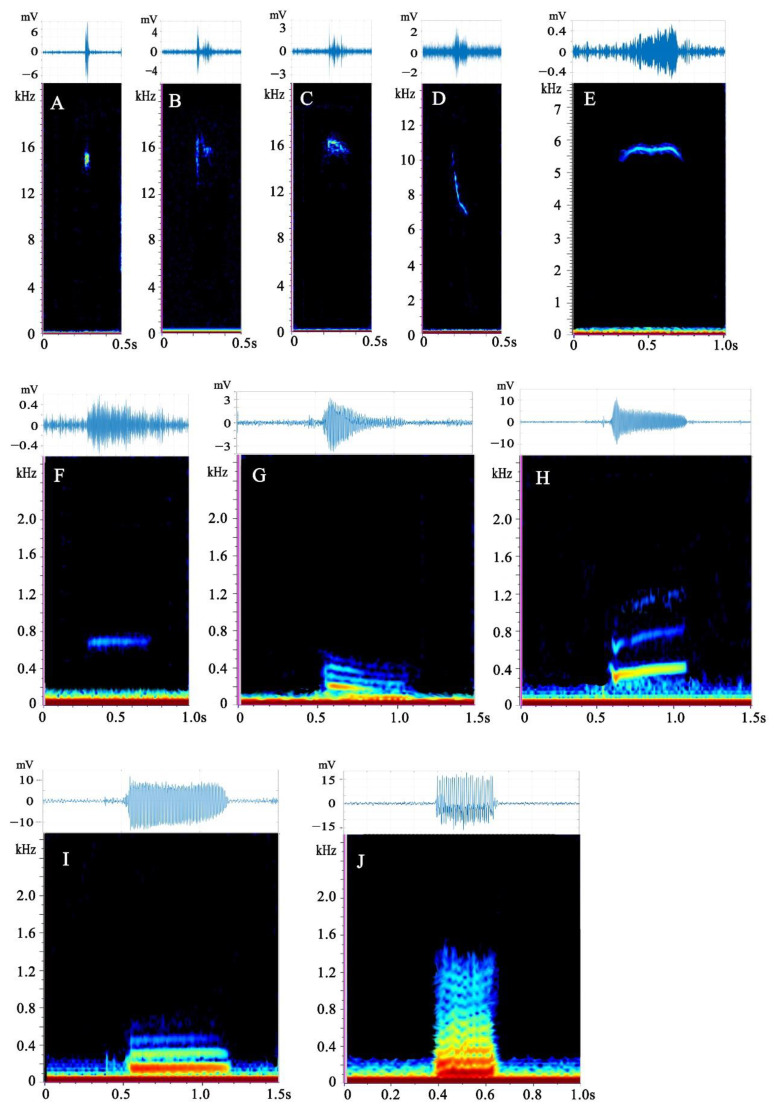
Spectrogram and waveform views of underwater sounds produced by *Pelodiscus sinensis*. All spectrograms were obtained with Raven Pro 1.5 using Hamming windows with 1024 pt fast Fourier transform. The waveforms were drawn in MATLAB R2021a. Capital letters A–J represent call types A–J.

We found a total of 720 calls, of which 143 were produced by subadult males, 117 by subadult females, 192 by adult males, 133 by adult females, 135 by mixed-sex adults, and none by juveniles (Table 1). No courtship behavior was detected during the recording of the mixed-sex adult group. Turtle calls were classified into 10 types (Table 1 and Table 2 and Figure 1). Audio samples of each type are available in Supplementary Audio A–J. Acoustical parameters for each call are shown in Supplementary Table S1.

### 3.1. Description of Call Types

Ten call types were identified, of which five were high-frequency calls and five were low-frequency calls. The statistics of their acoustic parameters are listed in Table 2 and their spectra are shown in Figure 1.

Type A was a high-frequency call. It is a short vertical line on the spectrum, looking like a pulse (Figure 1A) with a very short duration. It was the most common call type of the Chinese soft-shelled turtle; more than half of them were produced by subadults.

Type B was also a high-frequency call. The spectrum is composed of two parts: the front is a vertical pulse signal, followed by a point signal (Figure 1B). This call type can be found in all age and sex groups, and more than half were produced by subadult males.

Type C is a high-frequency call that has a fan-shaped spectrum (Figure 1C), and its peak frequency is stable at approximately 14 kHz; Approximately half of the calls were emitted by adult females, but very few by adult males.

Type D is a high-frequency call with an oblique vertical line on the spectrum, the top half of which is vertical and the bottom half rightward (Figure 1D). It has a wide bandwidth. More than half of the calls were emitted by subadult females but none by subadult males.

Type E is a medium-high frequency call that appears M-shaped or inverted V-shaped on the spectrogram, with one or three inflection points (Figure 1E). This call has a long duration. Half of the calls were produced by subadult males.

Type F is a low-frequency call with a long duration. It appeared as a straight line in the spectrum (Figure 1F). This type occurred in all age and sex groups, with adult females accounting for more than 30 % of the calls.

Type G is a low-frequency call with 1–11 down-sweep harmonics on the spectrogram (Figure 1G) for a long duration. The peak frequency is stable at approximately 100 Hz. More than two-thirds of calls were produced by adult males, but none by adult females.

Type H is a low-frequency call, and the spectrum diagram shows 1–7 up-sweep harmonics with a turning point (Figure 1H). More than half of these calls were produced by adult males.

Type I is a low-frequency multi-harmonic call, whose spectrum diagram is a parallel horizontal line with 1–7 harmonics, most are 2–3 harmonics (Figure 1I). Most calls of this type were produced by adults, with very few produced by subadults. This call type is the most common type of harmonic call in Chinese soft-shell turtles.

Type J is a low-frequency multi-harmonic call, and the spectrum diagram shows a curved shape with 2–12 harmonics and multiple inflection points (Figure 1J). Its peak frequency is 100 Hz. Only 10 calls of this type were found, all emitted by adults.

### 3.2. Similarity Calculation

All the correlation coefficients between the calls within each call type were larger than 0.6, averaging 0.73, which means that the similarity between calls in each call type was high, and the difference was small. All the correlation coefficients between the 10 call types were lower than 0.22, which means that the call similarity between each type was low, and the difference was large. Thus, the manual classification of calls by Chinese soft-shell turtles had high reliability. The specific correlation coefficients are presented in Table 3.

### 3.3. Differences in Vocalizations between Sexes and Age Groups

#### 3.3.1. Peak Frequency

**Figure 2:**
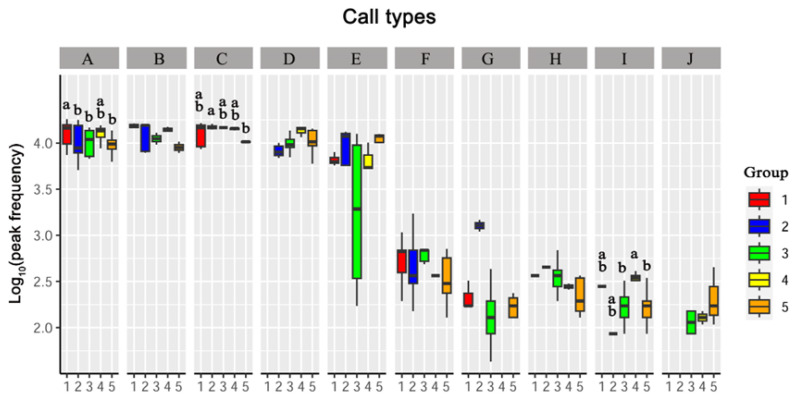
Differences analysis in peak frequencies between five groups. Capital letters A–J represent call types A–J. Boxplots with different lowercase letters indicate significant results (*p* < 0.05). Boxplots with the same lowercase letters indicate no significant difference (*p* > 0.05). When boxplots are marked with only one letter, the boxplot marked “a” is significantly higher than the boxplot marked “b” in a call type (*p* < 0.05). Call types without letters on the boxplots means no significant differences between groups. Groups: 1 represents subadult males, 2 represents subadult females, 3 represents adult males, 4 represents adult females and 5 represents mixed-sex adults.

Three of the call types (Types A, C, and I) showed significant differences in the peak frequencies among the five groups. There is no significant differences in their peak frequencies among groups in other call types (Figure 2; Supplementary Table S2).

In Type A, the peak frequency of subadult males showed significantly higher than that in the other groups (*p* < 0.05). No significant differences in peak frequency were detected between subadult females, adult males, and mixed-sex adults (*p* > 0.05).

In Type C, subadult females showed a significantly higher peak frequency than mixed-sex adults (*p* < 0.05). There were no significant differences among the subadult males, adult males, and adult females (*p* > 0.05).

In Type I, adult females showed significantly higher peak frequencies than adult males and mixed-sex adults (*p* < 0.05). There were no significant differences among subadult males, subadult females, adult males, and mixed-sex adults. (*p* > 0.05). In addition, there were no significant differences between the adult females and subadult groups (*p* > 0.05).

#### 3.3.2. Duration

**Figure 3:**
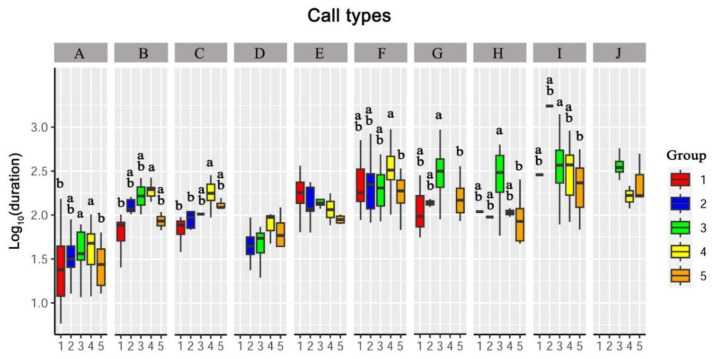
Differences analysis of call durations between five groups. Capital letters A–J represent call types A–J. Boxplots with different lowercase letters indicate significant results (*p* < 0.05). Boxplots with the same lowercase letters indicate no significant difference (*p* > 0.05). When boxplots are marked with only one letter, the boxplot marked “a” is significantly higher than the boxplot marked “b” in a call type (*p* < 0.05). Call types without letters on the boxplots means no significant differences between groups. Groups: 1 represents subadult males, 2 represents subadult females, 3 represents adult males, 4 represents adult females and 5 represents mixed-sex adults.

Seven call types (Types A, B, C, F, G, H, and I) showed significant differences in call duration among the five groups. (Figure 3; Supplementary Table S3).

In type A, adult females and males showed significantly longer call duration than subadult males and mixed-sex adults (*p* < 0.05). There were no significant differences between adult females and males, between subadult males, subadult females and mixed-sex adults (*p* > 0.05).

In Type B, the adult female group had a significantly longer call duration than the subadult male group (*p* < 0.05). No significant differences were detected between adult females and adult males or between subadult females and subadult males (*p* > 0.05).

In Type C, adult females showed significantly longer call durations than subadult males and subadult females (*p* < 0.05). There were no significant differences between adult females and adult males or between subadult females and subadult males (*p* > 0.05). Mixed-sex adults showed no significant differences from other groups (*p* > 0.05).

In Type F, adult females showed a significantly longer call duration than mixed-sex adults (*p* < 0.05). No significant differences were detected among the other groups (*p* > 0.05).

In Type G, adult males had a significantly longer call duration than mixed-sex adults (*p* < 0.05). No significant differences were detected among the other groups (*p* > 0.05).

In Types H and I, adult males presented significantly longer call durations than mixed-sex adults (*p* < 0.05). No significant differences were detected among the other groups (*p* > 0.05).

## 4. Discussion

### 4.1. Vocalization Diversity

The Chinese striped-neck turtle (*M. sinensis*), a sympatric species of the Chinese soft-shelled turtle, can produce 10 call types (three types are harmonics) [18]. Of these call types, only one is a high-frequency pulse call type. In contrast, Chinese soft-shelled turtle can produce four high-frequency pulse call types, with much higher peak frequencies than those of Chinese striped-neck turtles. Both species vocalize down-sweep harmonics, but the peak frequency and high frequency of the Chinese striped-neck turtle’s harmonics are higher than those of the Chinese soft-shelled turtle (the harmonics of the Chinese striped-neck turtle can reach 7 kHz, while the harmonics of the Chinese soft-shelled turtle are below 2 kHz). In addition, the Chinese striped-neck turtle has a low-frequency call type that is very similar to call type F of the Chinese soft-shelled turtle (spectrogram and auditory), but the peak frequency of Chinese soft-shelled turtles is approximately twice as high as that of Chinese striped-neck turtles. Whether the very similar vocalizations between the two species are due to their similar living environments and whether they have the same communication function requires further study.

Of the 12 call types produced by the red-eared turtle (*T. scripta elegans*), another sympatric species of Chinese soft-shelled turtle, none resemble those produced by the Chinese soft-shelled turtle [12]. Among the harmonic calls of long-necked turtles (*Chelodina oblonga*), three call types are very similar to call types G, I, and J of Chinese soft-shelled turtles [10]. Although the habitats of the two species do not overlap, they are both carnivorous and often dive into the sediments of water [10,25]. This finding may be explained by their similar lifestyles.

Many tortoises, freshwater turtles, and sea turtles can vocalize, and most have been shown to produce multi-harmonic calls [2,3,6,10,11,13,33]. Chinese soft-shelled turtles can produce 10 call types underwater, four of which are multi-harmonic calls. Many turtle species can produce multi-harmonic calls, which may be determined by the similar larynx structure of turtles [34]. The structure and position of the larynx of the turtle are similar to those of the mammalian vocal cords, and the two diverticula held by the cricoid might function as low-frequency resonating chambers, improving the harmonic structure of tortoise calls [21,35]. Similar to the Chinese soft-shelled turtle, other identified turtle species with more call types and more harmonic calls are highly aquatic species that prefer to live in deep water, such as *P. expansa* (11 call types, six of which are harmonics) and *C. oblonga* (17 call types, nine of which are harmonics) [10,36]. These species might have developed a greater variety to adapt to dim underwater environments.

The matched filter hypothesis proposes that tuning of the receiver’s auditory sensitivity will evolve to closely match the dominant frequency of species-specific calls [37]. Several studies on insects, fish, birds, and anurans have verified this hypothesis [38,39]. However, because of the influences of natural and sexual selection, a mismatched relationship might arise between the sender’s signals and the receiver’s auditory sensitivity [40,41]. Existing research has revealed that the hearing range of turtles is concentrated at low-frequencies [42,43,44,45]; however, aquatic turtle species have been found to produce high-frequency calls that can reach 6000–15,000 Hz [6,11,12,46]. The Chinese soft-shelled turtle can produce high-frequency calls underwater, similar to its sympatric red-eared turtle (*T. scripta elegans*) in China [12]. The advantage of high-frequency calls is that they help resist interference from low-frequency noise in communication, since natural waters are full of low-frequency noise [47]. For instance, frogs (*Odorrana tormota* and *O. graminea*) and birds (*Abroscopus albogularis*) inhabiting aquatic environments produce calls containing ultrasonic components to avoid masking by wideband noise [48,49,50]. As there have been no studies on the hearing threshold of Chinese soft-shelled turtles, we cannot be sure whether they can hear such high calls or whether these high-frequency calls have communication functions. In some reptiles, high-frequency calls are often used to intimidate heterospecific intruders but are not heard by conspecifics [51,52,53,54].

Vocalization has been found to change with age in bats (*Myotis nattereri bombinus* and *Rhinolophus ferrumequinum*) [55,56], and this kind of change for *Lonchura striata swinhoei* was shown to be associated with the development of neural modulation mechanisms [57]. The differences in vocalization and auditory sensitivity between Lusitanian toadfish (*Halobatrachus didactylus*) juveniles and adults demonstrates that acoustic communication may be absent in their early developmental stages [58]. As *Melopsitiacus undelafusp* grows, its calls become more complex and higher in amplitude; as they grow, male birds have more complex calls than female birds [59]. American Coots (*Fulica americana*) change their call types as they grow [60]. The harmonic number of the song of the lesser horseshoe bat (*R. cornutus*) increases with age after birth [61]. In rhesus monkeys (*Macaca mulatta*), the pitch of the “cooing” drops and the amplitude becomes more constant as they age [62]. Green sea turtle (*C. mydas*) hatchlings produce only four call types, whereas subadults can emit 11 call types [13]. In this study, juvenile Chinese soft-shelled turtles were not found to produce vocalizations; the subadults produced a large number of high-frequency calls, but only a very small number of low-frequency harmonic call types; and adults produce a more balanced number of different types of calls. This shows a tendency for the vocalization of Chinese soft-shelled turtles to become more diverse with age. Whether this phenomenon is related to the degree of development of the physiological function of the vocal organs requires further study.

### 4.2. Sex and Age Differences in Vocalization

Diverse vocalizations among individuals in an animal population are often caused by differences in the structure of the vocal organs [63,64]. A larger body size usually indicates a larger vocal organ that produces lower frequencies [65,66]. Many birds and frogs have verified that their dominant frequencies of vocalization are inversely related to body size [67,68,69]. The vocal frequency of tortoises is found inversely proportional to their size [4]. In our recordings, the Chinese soft-shelled subadults had higher peak frequencies and shorter durations in two high-frequency call types. This may be because the size of subadults is smaller than that of adults, or it may be due to age differences in the neural modulation of vocalizations [52].

However, the frequency of animal calls is not always inversely related to their body size. In several owl species, females produce higher calls than males, but females are larger than males [70]. Male baboons (*Papio cynocephalus*), gibbons (*Hylobates lar*), and chimpanzees (*Pan troglodytes*) are larger than their respective females but have higher peak frequencies than their females in some call types [71,72,73]. *Jacana spinosa* is larger than *J. jacana* but emits higher frequency calls than *J. jacana* [64]. Adult females of red-eared turtles (*T. scripta elegans*) are much larger than adult males of the same age but produce more high-frequency calls than adult males [12]. For call type I, which was the most frequent low-frequency call, the peak frequencies of adult females were significantly higher than that of adult males; however, the body sizes of the two groups were similar. For type A, there was also a difference in the peak frequency between male and female subadults of similar body size. Recent research has revealed that freshwater turtles show differences in sexually dimorphic hearing sensitivity [40], which is associated with the amount of gene expression in neural inhibitory pathways for hub genes related to the inner ear and tympanic membrane [74]. Hence, sex differences in hearing sensitivity and auditory genes may be one reason for sex differences in vocalization.

The potential function of turtle calls remains unclear, but they may be important for inter-specific communication [12]. Research has shown that individual-specific vocalizations are used for the individual recognition of sea turtles [13]. It must be noted that because these calls were recorded in an artificial environment, the sound descriptions might not completely reflect free-field recordings [10].

## 5. Conclusions

We identified 720 underwater calls of Chinese soft-shelled turtles in recordings and divided them into 10 call types. These results provide basic data for other bioacoustics and evolutionary studies of Chinese soft-shelled turtles. Chinese soft-shelled turtles have a high vocal diversity and many harmonic calls, indicating that they may have developed a greater variety to adapt to dim underwater environments. In addition, Chinese soft-shelled turtles show a tendency for vocalization to become more diverse with age. The peak frequency of calls in Chinese soft-shelled turtles was significantly different between adult females and males and between subadults and adults. These findings suggest that further studies on the ecology and anatomy of Chinese soft-shelled turtle should be conducted to explain the mechanisms behind these phenomena. Further behavioral and auditory studies are needed to determine the intra-specific communication function and ecological implications of Chinese soft-shelled turtle vocalizations.

## Figures and Tables

**Table 1:** Number of call types produced by *Pelodiscus sinensis* in each group.

Type	Adult Females	Ratio (%)	Adult Males	Ratio (%)	Subadult Females	Ratio (%)	Subadult Males	Ratio (%)	Mixed-Sex Adults	Ratio (%)	Sum
A	27	11.49	21	8.94	65	27.66	69	29.36	53	22.55	235
B	8	22.22	2	5.56	5	13.89	19	52.78	2	5.56	36
C	26	49.06	1	1.89	6	11.32	15	28.30	5	9.43	53
D	3	10.00	6	20.00	16	53.33			5	16.67	30
E	3	9.38	4	12.50	5	15.63	16	50.00	4	12.50	32
F	27	30.68	14	15.91	16	18.18	19	21.59	12	13.64	88
G		0.00	69	77.53	2	2.25	3	3.37	15	16.85	89
H	2	6.25	19	59.38	1	3.13	1	3.13	9	28.13	32
I	34	29.57	52	45.22	1	0.87	1	0.87	27	23.48	115
J	3	30.00	4	40.00					3	30.00	10
Total	133		192		117		143		135		720

Note: This ratio is equal to the number of calls of a call type made by a group divided by the total number of calls of that call type.

**Table 2:** Descriptive statistics of the acoustic parameters of all call types.

Type	Low Frequency Median ± IQR (Hz)	High Frequency Median ± IQR (Hz)	Peak Frequency Median ± IQR (Hz)	Duration Median ± IQR (ms)	Bandwidth (Hz)	No. of Harmonics
A	10,656 ± 6218	11,567 ± 6324	10,745 ± 6180	31.57 ± 30.45	689 ± 947	
B	13,230 ± 5811	15,749 ± 1929	14,729 ± 3553	95.58 ± 92.17	1497 ± 1975	
C	13,714 ± 2248	14,539 ± 1811	14,320 ± 2068	122.86 ± 108.89	538 ± 527	
D	8572 ± 6323	10,262 ± 5093	9055 ± 6013	49.15 ± 32.49	722 ± 1098	
E	6506 ± 5939	7086 ± 5836	6406 ± 3726	137.92 ± 102.55	367 ± 333	
F	331 ± 303.25	426 ± 334	366 ± 367	244.76 ± 186.78	86 ± 43	
G	86 ± 56	385 ± 228	129 ± 108	284.08 ± 240.57	129 ± 86	1~11
H	275 ± 162	721 ± 503	345 ± 210	182.43 ± 264.17	129 ± 183	1~7
I	151 ± 158	424 ± 405	215 ± 172	327.62 ± 275.51	86 ± 21	1~7
J	84 ± 45	1058 ± 1039	140 ± 54	231.95 ± 232.79	216 ± 215	2~12

Note: “High frequency” indicates the upper limit of the frequency for a call type; “low frequency” indicates the lower limit of the frequency for a call type; “IQR” is the interquartile range of the call type.

**Table 3:** Average value of correlation coefficients for calls within each call type and between call types.

	Call Types	A	B	C	D	E	F	G	H	I	J
Average value of correlation coefficients between call types	A	0.71									
	B	0.16	0.77								
	C	0.15	0.21	0.78							
	D	0.15	0.22	0.21	0.71						
	E	0.12	0.15	0.17	0.17	0.80					
	F	0.14	0.18	0.20	0.20	0.16	0.77				
	G	0.11	0.15	0.15	0.16	0.12	0.14	0.77			
	H	0.10	0.14	0.15	0.14	0.11	0.13	0.11	0.60		
	I	0.11	0.15	0.16	0.15	0.13	0.15	0.11	0.10	0.71	
	J	0.14	0.18	0.19	0.19	0.15	0.17	0.14	0.12	0.14	0.63

## Data Availability

The data set of vocal parameters of the turtles used in this study can be found in Supplementary Table S1.

## Supplementary Materials

The following supporting information can be downloaded at: https://www.mdpi.com/article/10.3390/ani13050812/s1, Table S1: Acoustic parameters of each call produced by Chinese soft-shelled turtles; Table S2: Difference analysis of peak frequencies for 10 call types between five groups (Kruskal-Wallis tests); Table S3: Difference analysis of call durations for 10 call types between five groups (Kruskal-Wallis tests); Audio A: Sample of call type A; Audio B: Sample of call type B; Audio C: Sample of call type C; Audio D: Sample of call type D; Audio E: Sample of call type E; Audio F: Sample of call type F; Audio G: Sample of call type G; Audio H: Sample of call type H; Audio I: Sample of call type I; Audio J: Sample of call type J.
