# Supplementary material for: Diversity of Underwater Vocalizations in Chinese Soft-Shelled Turtle (Pelodiscus sinensis)
Source: Animals (Basel). 2023 Feb 23;13(5):812. doi: 10.3390/ani13050812 (PMC10000088; doi:10.3390/ani13050812)
Supplement: Supplementary file 1 [file animals-13-00812-s001.zip › Supplementary Table S2.pdf]

Supplementary Table S2. Difference analysis of peak frequencies for 10 call types between five groups (Kruskal-Wallis tests)

Type	*p*-value	Adult males	Adult females	Subadult females	Subadult males	Mixed-sex
A	Adult males		0.836	0.478	1.000	0.059
	Adult females	0.836		1.000	0.014	1.000
	Subadult females	1.000	0.478		0.000	1.000
	Subadult males	0.014	1.000	1.000		0.000
	Mixed sex	1.000	0.059	0.000	0.000	
**Type**	***p*-value**	**Adult males**	**Adult females**	**Subadult females**	**Subadult males**	**Mixed-sex**
B	Adult males		1.000	1.000	0.242	1.000
	Adult females	1.000		1.000	0.607	1.000
	Subadult females	1.000	1.000		1.000	1.000
	Subadult males	0.607	0.243	1.000		0.239
	Mixed sex	1.000	1.000	1.000	0.239	
**Type**	***p*-value**	**Adult males**	**Adult females**	**Subadult females**	**Subadult males**	**Mixed-sex**
C	Adult males		1.000	1.000	1.000	0.249
	Adult females	1.000		1.000	1.000	0.510
	Subadult females	1.000	1.000		1.000	1.030
	Subadult males	1.000	1.000	1.000		0.285
	Mixed sex	0.510	0.249	0.030	0.285	
**Type**	***p*-value**	**Adult males**	**Adult females**	**Subadult females**	**Subadult males**	**Mixed-sex**
D	Adult males					
	Adult females					
	Subadult females	$H = 5.418, N = 30, df = 3, p = 0.144$				
	Subadult males					
	Mixed sex					
**Type**	***p*-value**	**Adult males**	**Adult females**	**Subadult females**	**Subadult males**	**Mixed-sex**
E	Adult males					
	Adult females					
	Subadult females	$H = 5.774, N = 32, df = 4, p = 0.217$				
	Subadult males					
	Mixed sex					
**Type**	***p*-value**	**Adult males**	**Adult females**	**Subadult females**	**Subadult males**	**Mixed-sex**
F	Adult males		0.176	1.000	0.484	1.000
	Adult females	0.176		1.000	1.000	0.194
	Subadult females	1.000	1.000		1.000	1.000
	Subadult males	1.000	0.484	1.000		0.481
	Mixed sex	0.194	1.000	1.000	0.481	
**Type**	***p*-value**	**Adult males**	**Adult females**	**Subadult females**	**Subadult males**	**Mixed-sex**
G	Adult males		–	–	–	–
	Adult females	–		0.076	1.000	0.394
	Subadult females	0.076	–		1.000	0.591
	Subadult males	1.000	–	1.000		1.000
	Mixed sex	0.394	–	0.591	1.000	
**Type**	***p*-value**	**Adult males**	**Adult females**	**Subadult females**	**Subadult males**	**Mixed-sex**
H	Adult males					
	Adult females					
	Subadult females	$H = 5.660, N = 32, df = 4, p = 0.226$				
	Subadult males					
	Mixed sex					
**Type**	***p*-value**	**Adult males**	**Adult females**	**Subadult females**	**Subadult males**	**Mixed-sex**
I	Adult males		0.000	0.063	1.000	0.000
	Adult females	0.000		1.000	1.000	1.000
	Subadult females	1.000	0.063		1.000	1.000
	Subadult males	1.000	1.000	1.000		1.000
	Mixed sex	1.000	0.000	1.000	1.000	
**Type**	***p*-value**	**Adult males**	**Adult females**	**Subadult females**	**Subadult males**	**Mixed-sex**
J	Adult males					
	Adult females					
	Subadult females	$H= 2.104, N =10, df= 2, p = 0.349$				
	Subadult males					
	Mixed sex					

Note: "$p < 0.05$" indicates that there is a significant difference between groups; otherwise, there is no significant difference between groups; "-" indicates that comparisons cannot be made because there are less than two individual calls of that type in a group. Types D, E, H and J were not conducted paired comparison because there is no significant difference in the five groups for these two call types ($p > 0.05$).
