# Supplementary material for: Diversity of Underwater Vocalizations in Chinese Soft-Shelled Turtle (Pelodiscus sinensis)
Source: Animals (Basel). 2023 Feb 23;13(5):812. doi: 10.3390/ani13050812 (PMC10000088; doi:10.3390/ani13050812)
Supplement: Supplementary file 1 [file animals-13-00812-s001.zip › Supplementary Table S3.pdf]

**Supplementary Table S3**. Difference analysis of call durations for 10 call types between five groups (Kruskal-Wallis tests)

| **Type** | ***p*-value** | **Adult males** | **Adult females** | **Subadult females** | **Subadult males** | **Mixed sex** |
|---|---|---|---|---|---|---|
| | Adult males | | 1.000 | 0.715 | 0.002 | 0.007 |
| | Adult females | 1.000 | | 1.000 | 0.011 | 0.034 |
| A | Subadult females | 1.000 | 0.715 | | 0.104 | 0.366 |
| | Subadult males | 0.011 | 0.002 | 0.104 | | 1.000 |
| | Mixed sex | 0.034 | 0.007 | 0.366 | 1.000 | |
| **Type** | ***p*-value** | **Adult males** | **Adult females** | **Subadult females** | **Subadult males** | **Mixed sex** |
| | Adult males | | 1.000 | 1.000 | 0.242 | 1.000 |
| | Adult females | 1.000 | | 1.000 | 0.607 | 1.000 |
| B | Subadult females | 1.000 | 1.000 | | 1.000 | 1.000 |
| | Subadult males | 0.607 | 0.243 | 1.000 | | 0.239 |
| | Mixed sex | 1.000 | 1.000 | 1.000 | 0.239 | |
| Type | ***p*-value** | Adult males | Adult females | Subadult females | Subadult males | Mixed sex |
| | Adult males | | 0.000 | 0.017 | 0.000 | 1.000 |
| | Adult females | 1.000 | | 1.000 | 1.000 | 1.000 |
| C | Subadult females | 1.000 | 0.017 | | 1.000 | 1.030 |
| | Subadult males | 1.000 | 0.000 | 1.000 | | 1.000 |
| | Mixed sex | 0.510 | 1.000 | 1.000 | 1.000 | |
| Type | ***p*-value** | Adult males | Adult females | Subadult females | Subadult males | Mixed sex |
| | Adult males | | | | | |
| | Adult females | | | | | |
| D | Subadult females | $H = 3.922, N = 30, df = 3, p = 0.270$ | | | | |
| | Subadult males | | | | | |
| | Mixed sex | | | | | |
| Type | ***p*-value** | Adult males | Adult females | Subadult females | Subadult males | Mixed sex |
| | Adult males | | | | | |
| | Adult females | | | | | |
| E | Subadult females | $H = 7.792, N = 32, df = 4, p = 0.099$ | | | | |
| | Subadult males | | | | | |
| | Mixed sex | | | | | |
| Type | ***p*-value** | Adult males | Adult females | Subadult females | Subadult males | Mixed sex |
| | Adult males | | 0.118 | 0.087 | 0.138 | 0.027 |
| | Adult females | 0.118 | | 1.000 | 1.000 | 1.000 |
| F | Subadult females | 1.000 | 0.087 | | 1.000 | 1.000 |
| | Subadult males | 1.000 | 0.138 | 1.000 | | 1.000 |
| | Mixed sex | 1.000 | 0.027 | 1.000 | 1.000 | |
| Type | ***p*-value** | Adult males | Adult females | Subadult females | Subadult males | Mixed sex |
| | Adult males | | – | – | – | – |
| | Adult females | – | | 0.491 | 0.156 | 0.001 |
| G | Subadult females | 0.491 | – | | 1.000 | 1.000 |
| | Subadult males | 0.156 | – | 1.000 | | 1.000 |
| | Mixed sex | 0.001 | – | 1.000 | 1.000 | |
| Type | ***p*-value** | Adult males | Adult females | Subadult females | Subadult males | Mixed sex |
| | Adult males | | 1.000 | 1.000 | 1.000 | 1.000 |
| | Adult females | 1.000 | | 1.000 | 1.000 | 0.011 |
| H | Subadult females | 1.000 | 1.000 | | 1.000 | 1.000 |
| | Subadult males | 1.000 | 1.000 | 1.000 | | 1.000 |
| | Mixed sex | 0.011 | 1.000 | 1.000 | 1.000 | |
| Type | ***p*-value** | Adult males | Adult females | Subadult females | Subadult males | Mixed sex |
| | Adult males | | 1.000 | 1.000 | 1.000 | 0.072 |
| | Adult females | 1.000 | | 1.000 | 1.000 | 0.014 |
| I | Subadult females | 1.000 | 1.000 | | 1.000 | 0.252 |
| | Subadult males | 1.000 | 1.000 | 1.000 | | 1.000 |
| | Mixed sex | 0.014 | 0.072 | 0.252 | 1.000 | |
| Type | ***p*-value** | Adult males | Adult females | Subadult females | Subadult males | Mixed sex |
| | Adult males | | | | | |
| | Adult females | | | | | |
| J | Subadult females | $H = 3.973, N = 10, df = 2, p = 0.137$ | | | | |
| | Subadult males | | | | | |
| | Mixed sex | | | | | |

Note: "$p < 0.05$" indicates that there is a significant difference between groups; otherwise, there is no significant difference between groups; "–" indicates that comparisons cannot be made because there are less than two individual calls of that type in a group. Types D, E and J were not conducted paired comparison because there is no significant difference in the five groups for these two call types ($p > 0.05$).
